# Exploring the effect of GenAI on learning outcomes in higher education: a three-level meta-analysis

**DOI:** 10.3389/fpsyg.2026.1758670

**Published:** 2026-05-15

**Authors:** Changxin Fan, Lele Ke, Zexiong Chen, Pin Lv

**Affiliations:** 1 Institute of Education, Xiamen University, Xiamen, China; 2 Center for Teaching and Learning Development, Xiamen University, Xiamen, China; 3 School of Marxism, NingboTech University, Ningbo, China; 4 School of Educational Science, Shenyang Normal University, Shenyang, China

**Keywords:** ChatGPT, generative artificial intelligence, learning outcomes, meta-analysis, teaching method

## Abstract

**Introduction:**

As generative artificial intelligence (GenAI) becomes increasingly integrated into higher education, greater clarity is needed regarding its impact on student learning.

**Methods:**

This study conducted a three-level meta-analysis of 36 empirical studies, synthesizing 132 effect sizes from 7,229 participants. Learning outcomes were classified using the Learning Outcomes Thematic Group (LOTG) framework, and seven study-level moderators were examined.

**Results:**

The results indicate a significant medium overall effect of GenAI on learning outcomes (*g* = 0.499). Stronger effects were found for understanding, cognitive and creative outcomes (*g* = 0.669) and higher-order learning (*g* = 0.504), with moderate effects for dispositions (*g* = 0.452) and attainments (*g* = 0.363). Evidence was insufficient for the Using and Membership/inclusion/self-worth outcome categories. Teaching method was the only significant moderator, with collaborative learning (*g* = 1.026) and blended learning (*g* = 0.633) yielding the strongest effects, while other moderators showed no significant influence.

**Discussion:**

These findings suggest that GenAI is most effective when embedded in interactive and collaborative pedagogies. The study introduces a GenAI-Learning Alignment Perspective and outlines implications for instructional design, assessment practices, and teacher professional development.

## 1 Introduction

As one of the most visible and widely adopted tools within GenAI, ChatGPT has quickly permeated higher education, especially in instructional and learning contexts. Within days of its release, it surpassed one million users, signaling strong student interest and institutional attention (Johnston et al., 2024). Recent survey data further illustrate this rapid uptake. Although 91% of university leaders believe GenAI will enhance and personalize learning, faculty adoption continues to lag that of students. Nearly half of leaders expect net institutional benefits, whereas a notable minority remain cautious (Watson and Rainie, 2025). These patterns indicate that GenAI is becoming increasingly embedded in university environments even as its pedagogical value continues to be discussed and negotiated.

Despite this rapid diffusion, the role of ChatGPT in higher education remains contested. Reviews warn that GenAI may undermine academic integrity, even as they acknowledge benefits for assessment and feedback (Cotton et al., 2024). Other scholars argue that responsible use rather than prohibition is essential for supporting meaningful learning (Yu, 2023). Empirical findings reflect similar tensions. Some studies report improvements in critical and creative thinking, problem-solving, and motivation (Essel et al., 2024; Hu, 2024). Others document null or negative effects (Escalante et al., 2023; Li and Ironsi, 2024; Kosar et al., 2024). Lim et al. (2023) note that GenAI is both disruptive and potentially enriching. Such divergence suggests that educational technologies often have multiple and context-dependent influences, which reinforces the need for systematic meta-analytic synthesis to clarify how GenAI affects teaching and learning.

Several meta-analyses have begun to address this need, with their design characteristics summarized in Table 1. Most reviews include all educational levels, leaving higher education where learners often possess greater autonomy relatively underexamined. Although existing reviews generally converge on a moderate positive effect of GenAI on learning outcomes, important limitations remain. Many studies rely on broad learning-outcome frameworks that obscure variation across different domains of learning. Considerable heterogeneity persists across findings, likely due to limited attention to instructional design variables such as teaching method and GenAI technology characteristics such as platform and model version. Conventional meta-analytic approaches also frequently overlook the statistical dependence of effect sizes that originate from the same study, which may bias estimates.

**Table 1:** Prior meta analysis of GenAI's impact on learning outcome.

Authors	Educational level	Learning outcome construct	Moderators examined	k/n
Gökoglu and Erdogdu (2025)	k-12, college	Learning performance (*g* = 0.689)	(1) educational level, (2) sample size, (3) research design, (4) learning domain, (5) research setting, (6) intervention duration^*^, (7) GenAI tool, (8) testing format^*^	33/31
Gu and Yan (2024)	k-12, college	Academic achievement (*g* = 0.683)	(1) GenAI tool, (2) teacher support^*^, (3) intervention duration, (4) educational level, (5) discipline, (6) control group feedback, (7) sample size, (8) study quality,	24/19
Liu et al. 2025a)	k-12, college	Academic achievement (*g* = 0.577)	(1) educational level, (2) discipline^*^, (3) intervention duration^*^, (4) sample size^*^, (5) learning approach, (6) generate content^*^, (7) knowledge type^*^, (8) role setting, (9) instructional model^*^	37/37
Liu et al. 2025b)	k-12, college	Learning achievement (*g* = 0.857) Learning motivation (*g* = 0.803)	(1) educational level, (2) classification of subject^*#^, (3) GenAI interface, (4) GenAI development, (5) interaction approaches, (6) duration of experiment^*#^	79/49
Zhu et al. (2025)	k-12, college	Overall learning outcome (*g* = 0.392)	(1) functional types of GenAI, (2) educational levels, (3) intervention duration, (4) knowledge domains^*^	44/26
Sun and Zhou (2024)	college-only	academic achievement (*g* = 0.533)	(1) intervention duration, (2) discipline types, (3) assessment tools, (4) generate content, (5) sample size, (6) activity categories	65/28

(1) k denotes the number of effect sizes, and n denotes the number of studies included; (2) ^*^ indicates a significant moderating effect, ^#^ indicates a significant moderator for learning motivation in Liu et al. (2025b).

To address these gaps, this study conducts a three-level meta-analysis of GenAI effects on university learning outcomes. Using the Learning Outcomes Thematic Group (LOTG) framework, outcomes are classified into six domains to capture meaningful differences. By nesting effect sizes within samples and studies, the analysis retains information while appropriately modeling statistical dependence. The study also examines theoretically grounded moderators, including teaching method, GenAI platform, and model version. The goals are to estimate both overall and domain-specific associations between GenAI and higher-education learning outcomes, to identify conditions that strengthen or weaken these effects, and to derive implications for instructors, instructional designers, and policymakers.

## 2 Literature review

### 2.1 Conceptual framework of learning outcomes

Over recent decades, learning outcomes have become an increasingly prominent focus in higher education, not only because they signal what students gain from instruction but also because they inform how learning is designed, assessed, and supported (Hussey and Smith, 2008; Caspersen and Frølich, 2017). As research expands—particularly with the rise of GenAI in learning environments—the diversity of outcomes under investigation makes it necessary to classify them in a structured way so that findings can be meaningfully compared and interpreted. To provide such structure, prior work has often relied on Bloom's taxonomy and its three domains—cognitive, affective, and psychomotor/behavioral (Varela, 2017; Doo et al., 2023). However, as GenAI-enabled learning increasingly involves complex processes such as performance, motivation, self-efficacy, and critical thinking, recent syntheses have moved beyond Bloom's domains (Wu and Yu, 2024; Liu et al., 2025b), suggesting that the original tripartite model no longer fully reflects the breadth of outcomes now examined in AI-mediated contexts. In response to this expanded landscape, the present study adopts the LOTG framework (James and Brown, 2005), which organizes outcomes into six categories: (a) attainment, (b) understanding/cognitive/creative, (c) using, (d) higher-order learning, (e) dispositions, and (f) membership/inclusion/self-worth. This framework provides a more comprehensive and practical basis for coding GenAI's effects on learning outcomes in higher education. The six categories, their definitions, and examples from the included studies are summarized in Table 2.

**Table 2:** Learning outcome categories, definitions, and representative examples.

Category	Adapted definition (examples from the included studies)
Attainments	Mastery of specific knowledge, facts, or procedures, usually demonstrated through scores on tests, exams, or standardized tasks. Focuses on content mastery and accuracy. (e.g., exam scores; speaking fluency/accuracy; writing scores)
Understanding/ cognitive/ creative	Conceptual grasp of ideas, reasoning within domains, and creative integration of knowledge into artifacts or projects. Emphasizes depth of understanding and innovative use of knowledge. (e.g., clinical reasoning; project design performance; narrative intelligence)
Using	Application of knowledge and skills in hands-on, practical, or task-based contexts. Emphasizes doing, performing, or applying knowledge in real or simulated settings. (e.g., programming tasks; hardware operation performance; project performance)
Higher-order learning	Advanced thinking processes that go beyond recall or application, including critical thinking, reflective thinking, metacognition, self-regulation, and problem solving. (e.g., critical thinking; problem-solving ability; self-regulated learning strategies)
Dispositions	Learners' motivational, attitudinal, and emotional orientations toward learning, which influence their willingness to engage effectively. (e.g., intrinsic motivation; self-efficacy; learning attitudes)
Membership/ inclusion/ self-worth	Learners' sense of belonging, social connection, and perceived value within a learning community. (e.g., empathy; willingness to communicate; perceived communicative competence)

### 2.2 Effects of GenAI on learning outcomes

Existing studies on the effects of GenAI on learning outcomes report mixed findings across outcomes, with some documenting clear benefits and others showing limited or no impact.

#### 2.2.1 Attainment

Positive evidence shows that GenAI can improve programming knowledge, academic writing scores, and STEM teaching literacy (Huang et al., 2023; Ji et al., 2023; Mahapatra, 2024). By contrast, other studies find no significant effect on English speaking performance, nursing knowledge, or writing performance (Escalante et al., 2023; Chen et al., 2025; Hu et al., 2025).

#### 2.2.2 Understanding/Cognitive/Creative

Several studies report that GenAI enhances conceptual understanding, domain-specific reasoning, and creative design projects such as game-based health promotion and instructional courseware (Li, 2023; Hu, 2024; Wang et al., 2025). In contrast, no significant improvement was observed in students' clinical reasoning competency (Han et al., 2022).

#### 2.2.3 Using

Positive effects are reported for hands-on tasks, including AIoT hardware operation, hardware programming, and Arduino development projects (Wang et al., 2025; Lin et al., 2024). However, in other cases, such as a radar-chart programming assignment, GenAI did not yield significant benefits (Sun et al., 2024).

#### 2.2.4 Higher-order learning

Evidence indicates that GenAI can promote critical thinking (Chang et al., 2024a; Yilmaz and Yilmaz, 2023) and creative thinking (Habib et al., 2024), and also support gains in metacognition (Huang et al., 2023; Essel et al., 2024). Yet other research shows no improvement in critical or creative thinking (Lu et al., 2024) and reports mixed or even negative findings for aspects of self-regulated learning (Han et al., 2022; Pan et al., 2025).

#### 2.2.5 Dispositions

GenAI-supported instruction often enhances intrinsic motivation, self-efficacy, and learning attitudes (Li, 2023; Huang, 2024; Chang et al., 2025). Nevertheless, no significant changes are observed in extrinsic motivation, test anxiety, confidence, or interest (Han et al., 2022; Chen et al., 2025).

#### 2.2.6 Membership/inclusion/self-worth

Positive findings suggest that GenAI enhances empathy among nursing students (Chang et al., 2024b, 2025). Conversely, other studies report no significant effect on self-perceived communicative competence (Wang et al., 2024).

Given these inconsistencies, a meta-analytic approach is needed. Such an approach can estimate GenAI's overall and category-specific effects on learning outcomes and examine the moderating variables that account for heterogeneity, thereby offering guidance on when and how GenAI can be most effectively used in higher education practice.

### 2.3 Potential moderators

The effect of GenAI on college students' learning outcomes may be influenced by several moderators, yet the literature lacks consensus on which factors matter most. Existing research has largely examined situational moderators—such as discipline category, measurement tool, and intervention duration—reflecting a common effort to compare outcomes across different educational contexts. However, such contextual variables alone offer limited explanatory power for why GenAI produces divergent effects, since GenAI is an interactive, generative, and dynamically evolving intelligent system whose influence depends not only on the setting in which it is deployed but also on how it is used, the nature of the learning task, and specific technological attributes. Consequently, beyond retaining the three commonly analyzed moderators to ensure conventionality and comparability, this study extends prior work by examining four additional moderators—teaching method, learning task type, GenAI platform, and GenAI model version—to capture pedagogical and technological conditions that more directly shape GenAI's impact in higher education. This broader scope aims to yield more actionable insights for practice and policy.

#### 2.3.1 Teaching method

Educational theories posits that instructional method influences learning outcomes more directly than the medium of delivery. Clark (1994) argued that media may alter cost or speed but achievement is determined by method, a point later debated in the Clark–Kozma exchange. As the newest form of educational media, GenAI does not automatically guarantee improvement, and the pedagogical strategy guiding its use is determinant of its impact. GenAI can provide explanations, worked examples, formative feedback, or peer-like dialogue, yet their value depends on how they are embedded in learning activities. Methods that promote interactive and constructive engagement, such as collaborative critique or student generation with feedback, are theorized to yield stronger effects than passive uses (Chi and Wylie, 2014). Moreover, feedback quality and timeliness, often strengths of GenAI, are most effective when aligned with explicit goals and criteria (Hattie and Timperley, 2007; Shute, 2008). Accordingly, treating teaching method as a moderator is theoretically warranted, as variations in scaffolding, interaction, and cognitive engagement embedded in different methods may amplify or constrain GenAI's educational impact.

#### 2.3.2 Learning task type

It captures the fundamental orientation of an instructional activity. According to Anderson's ACT-R theory of skill acquisition, which distinguishes declarative from procedural knowledge (Anderson, 1982, 1983), tasks can be classified into two types: knowledge acquisition, oriented toward developing declarative understanding, and problem resolution, requiring procedural application and problem solving. This distinction is further reinforced by cognitive load research showing that guidance is most beneficial during acquisition, whereas problem solving becomes more effective as knowledge proceduralizes—the “expertise-reversal” pattern—suggesting that tool effectiveness is contingent on task type (Sweller and Cooper, 1985; Kalyuga et al., 2003).

#### 2.3.3 GenAI platform

Since the release of ChatGPT-3.5 in late 2022, generative AI has attracted unprecedented attention and triggered the proliferation of competing platforms. Different platforms vary in their underlying architectures, training data, alignment strategies, and interface designs, which shape the instructional affordances they provide (Norman, 2013). For example, user-facing features such as feedback framing, examples, or code sandboxes may influence learners' engagement, error regulation, and motivational persistence. Consequently, the educational impact of GenAI is unlikely to be uniform across platforms, making GenAI platform a theoretically meaningful moderator.

#### 2.3.4 GenAI model version

Since late 2022, successive model generations (e.g., from GPT-3.5 to GPT-4) have demonstrated marked improvements in reasoning and benchmark performance (OpenAI, 2023). Although technical advances do not guarantee proportional educational benefits, it is reasonable to expect that upgrades in model capacity could influence the extent to which GenAI supports learning in higher education (Nori et al., 2023). Version differences are therefore a theoretically warranted moderator, and to date no meta-analysis has systematically examined whether technological iterations shape the impact of GenAI on learning outcomes.

## 3 Method

### 3.1 Literature search strategy

Following the PRISMA 2020 guidelines, we searched the Web of Science (WoS) Core Collection, ERIC, Scopus, and China National Knowledge Infrastructure (CNKI) for relevant studies published between 30 November 2022, the public release date of ChatGPT, and 30 July 2025. The search strategies used in each database are presented below.

(1) WoS Core Collection: TS = (“generative artificial intelligence” OR “generative AI” OR “GenAI” OR “large language model^*^” OR LLM^*^) AND TS = (“learning outcome^*^” OR “student outcome^*^” OR “academic achievement” OR “learning performance” OR “academic performance”) AND DOP = (2022-11-30/2025-07-30).
(2) ERIC: TX (“generative artificial intelligence” OR “generative AI” OR GenAI OR “large language model^*^” OR LLM^*^ OR ChatGPT) AND TX (“learning outcome^*^” OR “student outcome^*^” OR “academic achievement” OR “learning performance” OR “academic performance” OR “learning gain^*^” OR “student learning”) AND TX (educat^*^ OR teach^*^ OR student^*^ OR “higher education” OR universit^*^ OR college^*^).
(3) CNKI: An advanced search was conducted in CNKI, using “生成式人工智能” in the Subject field and “实验组” in the Full-text field.
(4) Scopus: TITLE-ABS-KEY ((“generative artificial intelligence” OR “generative AI” OR GenAI OR “large language model^*^” OR LLM^*^) AND (“learning outcome^*^” OR “student outcome^*^” OR “academic achievement” OR “learning performance” OR “academic performance”)).

A total of 2,053 records were identified through database searching, including 691 from WoS, 253 from ERIC, 60 from CNKI, and 1,049 from Scopus.

### 3.2 Inclusion and exclusion criteria

**Figure 1:**
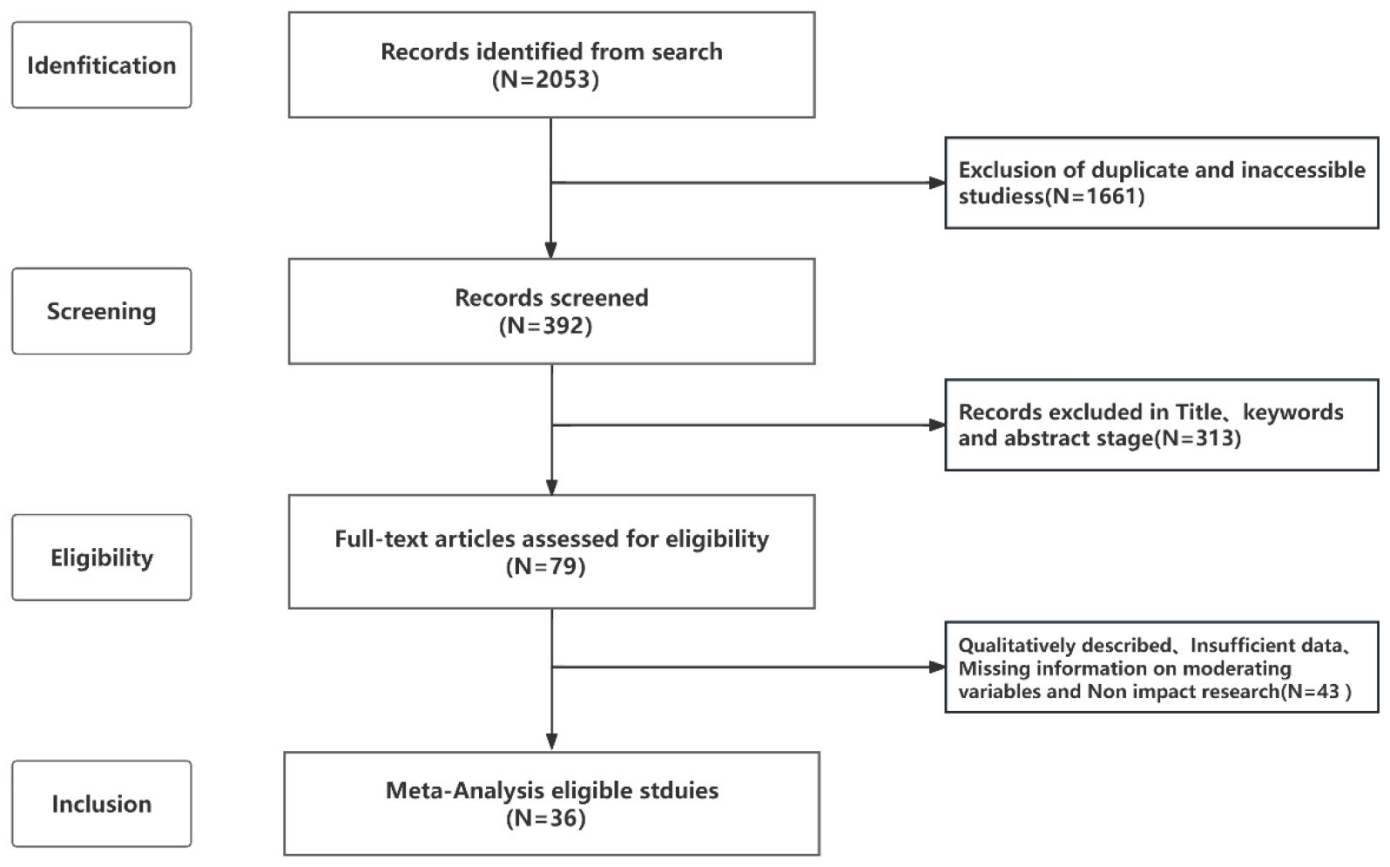
Study selection process following PRISMA 2020.

The PRISMA flowchart (Figure 1) illustrates the processes of identification, screening, eligibility assessment, and final inclusion in this study. In total, 2,053 records were identified through database searching. All search results were imported into EndNote for reference management and de-duplication. Duplicate records were first identified automatically using EndNote and then manually verified by comparing titles, authors, publication years, and journal names to ensure that multiple records from the same study were accurately removed. Records with inaccessible full texts were also excluded.

Subsequently, two rounds of screening were conducted. In the first stage, titles, abstracts, and keywords were screened to exclude studies that did not address (a) generative AI, (b) higher education, or (c) measurable learning outcomes. In the second stage, 79 full-text articles were assessed for eligibility against three inclusion criteria: (1) the study examined the impact of generative AI on learning outcomes in higher education; (2) it employed an experimental or quasi-experimental design comparing a GenAI group with a non-GenAI control group, including pre-post designs with a control group; and (3) it reported sufficient quantitative information, such as means, standard deviations, sample sizes, to compute effect sizes. After the second-stage screening, 36 studies were retained for the meta-analysis, contributing 132 effect sizes from a total of 7,229 participants.

### 3.3 Coding procedures and results

To examine potential sources of heterogeneity, we coded seven study-level moderators. Table 3 presents the coding results for each moderator, whereas Appendix A reports the full coding results for all 36 studies and 132 effect sizes. The coding process was completed independently by two authors, and the inter-rater reliability was substantial (Cohen's *k* = 0.832). Because the moderators differed in the extent to which they required interpretive judgment, their coding procedures are reported with different levels of detail below.

**Table 3:** Moderator coding results.

Moderator	Coding results
Discipline category	Humanities science, Natural science, Social science
Measurement tool	Expert rating, Self-rated scale, Standardized testing
Intervention duration	<1 week, 1–5 weeks, 6–10 weeks, 11–15 weeks, >15 weeks
Teaching method	Blended learning, Collaborative learning, Inquiry learning, Personalized Learning, Traditional teaching
Learning task type	Knowledge acquisition, Problem resolution
GAI platform	ChatGPT, Other
GAI model version	ChatGPT-3.5, ChatGPT-4.0
Study quality	High, Medium, Low

#### 3.3.1 Discipline category, measurement tool, and intervention duration

These moderators involved relatively low-inference coding because the relevant information was typically reported explicitly in the primary studies. Therefore, these moderators were coded directly based on study characteristics, following groupings commonly adopted in recent syntheses on the effects of educational technology on learning outcomes. The coding results for these moderators are shown in Table 3.

#### 3.3.2 Learning task type

Learning task type was coded into two categories: knowledge acquisition and problem resolution, as discussed in the literature review. Coding was based on the primary task reflected in the learning activities. Studies were coded as knowledge acquisition when the main task focused on understanding, explaining, summarizing, or applying existing knowledge in relatively well-defined ways. They were coded as problem resolution when the primary task emphasized solving problems, generating solutions, making decisions, or addressing open-ended challenges. When task features overlapped, coding was based on the task type that played the most central role in structuring the learning activities.

#### 3.3.3 Teaching method

In addition, teaching method was identified as particularly consequential because existing research suggests that learning effects depend more on pedagogy than on the medium of delivery. As no established external framework provides a definitive classification of GenAI-related teaching methods, we adopted a consistent pedagogy-based coding strategy. Each study was assigned to the single category that most accurately captured the dominant instructional logic reflected in its learning activities, rather than the delivery medium.

Teaching method was coded according to the dominant pedagogical logic reflected in the learning activities. In assigning categories, we considered three aspects in combination: (a) the core learning activity required of students, (b) the role of the instructor in organizing and guiding learning, and (c) the main process through which learning was expected to occur. When multiple pedagogical features were present, coding was based on the feature that played the most central role in structuring the learning activities and shaping student engagement. The classification focused on the underlying pedagogical orientation rather than surface features such as the technological format or delivery setting.

When category boundaries were potentially overlapping, the following decision rules were applied. Studies were coded as collaborative learning when peer coordination, shared task completion, or joint knowledge construction constituted the central learning mechanism. They were coded as inquiry learning when exploration, investigation, discovery, or problem solving was the primary mode of learning. They were coded as personalized learning when individual adaptation, self-paced learning, self-regulation, or tailored feedback formed the main pedagogical emphasis. They were coded as blended learning when the defining feature was the structured integration of online and face-to-face learning. Studies were coded as traditional teaching when learning was predominantly teacher-directed and content-transmissive, with students mainly receiving information rather than actively co-constructing or independently exploring knowledge.

Based on established educational frameworks (such as social constructivism, experiential learning, self-regulation theory, and blended learning models), the teaching methods in the included studies were clustered into five mutually exclusive groups:

(1) Collaborative learning. This cluster refers to pedagogies that emphasize peer interaction and joint knowledge construction, where students learn by coordinating and co-producing outcomes. Examples drawn from the included studies (all subsequent examples are also drawn from the included studies) include online collaborative writing, co-regulative learning, and group-based projects;
(2) Inquiry learning. This cluster covers pedagogies that promote inductive, learner-centered exploration and discovery, requiring students to construct knowledge through active investigation. Examples include inquiry-based teaching, guided discovery, role-playing, game-based learning, and VR-supported problem solving;
(3) Personalized learning. This category captures pedagogies designed to adapt learning processes to individual needs, fostering autonomy and self-regulation. Examples include practices such as self-regulated learning strategies and personalized feedback;
(4) Blended learning. This cluster denotes pedagogies that integrate technology-mediated and face-to-face instruction in a complementary way that alters when, where, or how learning occurs. Examples include flipped classrooms, mobile-assisted blended learning, and online–offline hybrids;
(5) Traditional teaching. This category refers to teacher-centered, content-transmission pedagogies, in which knowledge is delivered mainly through lectures or textbooks, with limited student interaction or autonomy. Students act primarily as passive recipients focusing on memorization and factual recall.

#### 3.3.4 GenAI platform and GenAI model version

GenAI platform and GenAI model version also involved relatively low-inference coding because the relevant information was generally reported explicitly in the primary studies. GenAI platform was coded into two categories: ChatGPT and other. This classification was adopted because ChatGPT was used in the majority of included studies (69.3%) and thus provided a meaningful basis for comparison. In addition, GenAI model version was coded as ChatGPT-3.5 and ChatGPT-4.0, as these were the most consistently reported versions in the included studies. By contrast, studies using other GenAI tools involved a wide variety of platforms, which did not allow for a consistent classification of model versions.

#### 3.3.5 Study quality

To address study quality concerns, this review conducted a methodological quality appraisal of the included studies. Drawing on the core logic of the EPHPP guideline and taking into account the characteristics of educational experimental and quasi-experimental research (Thomas et al., 2004), five domains were evaluated: (1) research design, (2) baseline equivalence between groups, (3) outcome measurement quality, (4) attrition and data completeness, and (5) intervention implementation and statistical analysis. Each domain was rated as high, medium, or low quality. The overall study quality rating was determined according to the number of domains rated as low: studies with no low-rated domains were classified as high quality; those with one low-rated domain were classified as medium quality; and those with two or more low-rated domains were classified as low quality. Study quality was used only for sensitivity analysis and was not included as a moderator in the moderator analyses.

### 3.4 Calculation of the effect size

Effect sizes were expressed as standardized mean differences (SMDs), and Hedges' *g* was used in this study. Among the SMD indices commonly used in educational research—Cohen's *d*, Glass's Δ, and Hedges' *g*—Hedges' g was selected because it applies a correction factor to the standardized mean difference to reduce small-sample bias (Hedges, 1981). Given the relatively small sample sizes in some included studies, Hedges' *g* was considered the most appropriate effect size measure for this meta-analysis.

For pretest-posttest-control studies, effect sizes were calculated as the difference in mean change between the intervention and control groups, standardized by the pooled pretest standard deviation, and then corrected for small-sample bias to obtain Hedges' *g*. Following Morris' (2008) approach for pretest-posttest-control designs, the mean change score in each group was first computed as:


$$\begin{array}{l} \Delta_{T}=M_{T,post}-M_{T,pre}\\ \Delta_{C}=M_{C,post}-M_{C,pre} \end{array}$$

where Δ_*T*_ is the mean change in the treatment group, Δ_*C*_ is the mean change in the control group, *M*_*T,post*_ and *M*_*T,pre*_ are the posttest and pretest means of the treatment group, and *M*_*C,post*_ and *M*_*C,pre*_ are the posttest and pretest means of the control group


$$\begin{array}{l} SD_{pre,pooled}=\sqrt{\frac{\left(n_{T}-1\right)SD_{T,pre}^{2}+\left(n_{C}-1\right)SD_{C,pre}^{2}}{n_{T}+n_{C}-2}} \end{array}$$

where *SD*_*T,pre*_ and *SD*_*C,pre*_ are the pretest standard deviations of the treatment and control groups, and *n*_*T*_ and *n*_*C*_ are the corresponding sample sizes.

The standardized mean difference for the pretest-posttest-control design was calculated as:


$$\begin{array}{l} d_{ppc}=\frac{\Delta_{T}-\Delta_{C}}{SD_{pre,pooled}} \end{array}$$

Finally, *d*_*ppc*_ was converted to Hedges' *g* using the small-sample correction:


$$\begin{array}{r} g=J\times d_{ppc}\\ J=1-\frac{3}{4df-1},df=n_{T}+n_{C}-2 \end{array}$$

When change-score standard deviations were required for variance estimation but were not reported in the primary studies, they were estimated using the pretest and posttest standard deviations together with an imputed pre-post correlation coefficient:


$$\begin{array}{l} SD_{change}=\sqrt{SD_{pre}^{2}+SD_{post}^{2}-2r\left(SD_{pre}\right)\left(SD_{post}\right)} \end{array}$$

where *SD*_*change*_ is the standard deviation of the change score, *SD*_*pre*_ is the pretest standard deviation, *SD*_*post*_ is the posttest standard deviation, and *r* is the pre-post correlation. In the present study, *r* = 0.50 was used when the pre-post correlation was unavailable (Higgins et al., 2019). Sensitivity analyses were additionally conducted using *r* = 0.40 and *r* = 0.60 to examine the robustness of the results.

### 3.5 Data analysis

Because many primary studies reported multiple eligible effect sizes from the same sample, a three-level meta-analysis was employed to account for statistical dependence and the hierarchical structure of the data. In this study, when a study reported multiple eligible outcomes, these outcomes were treated as conceptually distinct dimensions of learning outcomes rather than repeated measurements of the same endpoint. Accordingly, all eligible outcome dimensions were retained as separate effect sizes. These effect sizes were not assumed to be statistically independent; instead, their dependency was modeled directly in the three-level meta-analysis by specifying effect sizes from the same study as belonging to the same cluster. This approach allowed multiple eligible outcomes from the same study to be retained without analyzing them as independent observations. Formally, the model can be written as ${\hat{\theta }}_{ij}=\mu +\xi_{ij}^{\left(2\right)}+\xi_{j}^{\left(3\right)}+\varepsilon_{ij}$, where ${\hat{\theta }}_{ij}$ is the observed effect size *i* nested within study *j*; μ is the overall mean effect; ε_*ij*_ denotes the sampling error (Level 1); $\xi_{ij}^{\left(2\right)}$ represents the within-study heterogeneity (Level 2); and $\xi_{j}^{\left(3\right)}$ captures the between-study heterogeneity (Level 3; Cheung, 2014). Compared with the conventional two-level random-effects model, this specification reduces unit-of-analysis errors by allowing for dependence among multiple effects from the same study while still estimating an overall average effect.

Variance components and overall effects were estimated using restricted maximum likelihood (REML) via the metafor (Viechtbauer, 2010) and meta (Balduzzi et al., 2019) packages in R. To assess potential publication bias, funnel plots and Egger's regression test were conducted. Sensitivity analyses were then performed in three ways: (1) outlier diagnostics were conducted to identify effect sizes or studies that exerted disproportionate influence on the results; (2) the robustness of the findings was examined by varying the pre-post correlation coefficient (*r*) used in the variance estimation for pretest-posttest-control studies; (3) sensitivity analyses were conducted based on study quality by excluding studies rated as low quality. A two-tailed *p* < 0.05 was used as the threshold for statistical significance.

## 4 Results

### 4.1 Overall and category-specific effect

The present study employed a three-level random-effects meta-analysis to estimate the impact of GenAI tools on learning outcomes in higher education (*n* = 36 studies, *k* = 132 effect sizes). As shown in Table 4, the overall effect size was *g* = 0.499 (SE = 0.064, 95% CI [0.372, 0.627], *p* < 0.0001), indicating a statistically significant and medium positive effect according to Cohen's (2013) benchmarks. When broken down by outcome categories, GenAI demonstrated significant positive effects on attainments (*g* = 0.363), dispositions (*g* = 0.452), higher-order learning (*g* = 0.504), and understanding/cognitive/creative outcomes (*g* = 0.669), all with *p* < 0.05. In contrast, effects for membership/inclusion/self-worth (*g* = 1.328) and using (*g* = 0.662) were not statistically significant, as their confidence intervals included zero. These non-significant results are likely attributable to the small number of effect sizes available for these categories (both with *k* = 3).

**Table 4:** Overall and category-specific effect of GenAI on learning outcome.

Construct	*k*	*g*	95% CI		SE	*t*	*p*
			Lower	Upper			
Attainments	23	0.363	0.115	0.611	0.120	3.031	0.0061
Dispositions	34	0.452	0.230	0.674	0.109	4.146	0.0002
Higher-order learning	43	0.504	0.309	0.700	0.097	5.202	<0.0001
Membership/Inclusion/self-worth	3	1.328	−0.017	2.673	0.313	4.248	0.051
Understanding/cognitive/creative	26	0.669	0.366	0.973	0.148	4.539	0.0001
Using	3	0.662	−0.678	2.002	0.312	2.126	0.1674
Learning outcome	132	0.499	0.372	0.627	0.064	7.756	<0.0001

**Figure 2:**
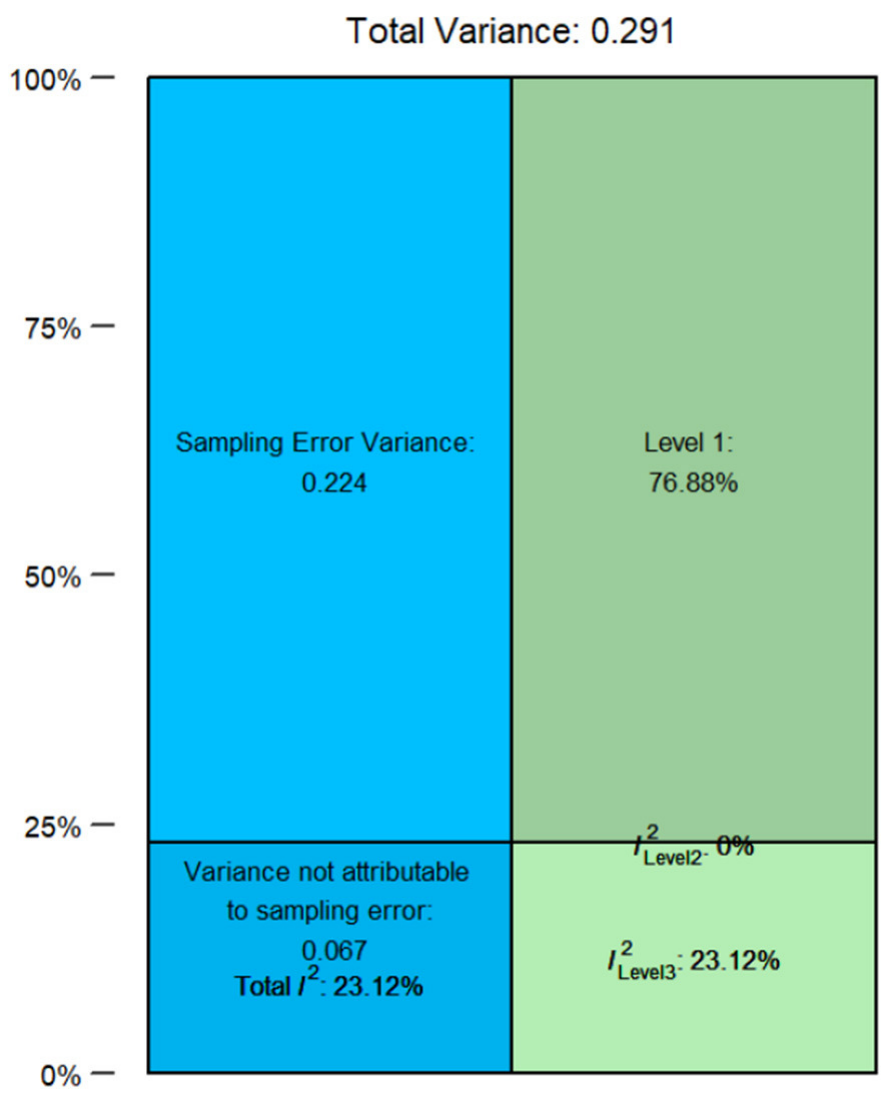
Heterogeneity distribution.

Working within the three-level framework, we decomposed the total variance into sampling error (Level 1), residual heterogeneity among effect sizes from the same study (Level 2), and heterogeneity between studies (Level 3; Cheung, 2014, 2019). As shown in Figure 2, the estimated variance components were τ^2^_Level 3 = 0.07 and τ^2^_Level 2 = 0, corresponding to *I*^2^_Level 3 = 23.12% and *I*^2^_Level 2 = 0. This pattern indicates that the observed heterogeneity was concentrated primarily at the between-study level, while the residual heterogeneity among multiple effect sizes from the same study was estimated at the boundary of zero. This should not be interpreted as evidence that within-study variation was literally absent; rather, it suggests that, after sampling error was taken into account, effect sizes derived from the same study were highly similar. Substantively, the remaining heterogeneity is therefore more likely to reflect differences across studies in participant characteristics, disciplinary contexts, intervention duration, teaching methods, and measurement tools. Because the effect sizes were still nested within studies, we retained the three-level specification. Based on Occam's razor, we compared a two-level with the full three-level model. a likelihood-ratio test favored retaining the three-level structure (LRT = 11.523, *p* = 0.0007).

**Figure 3:**
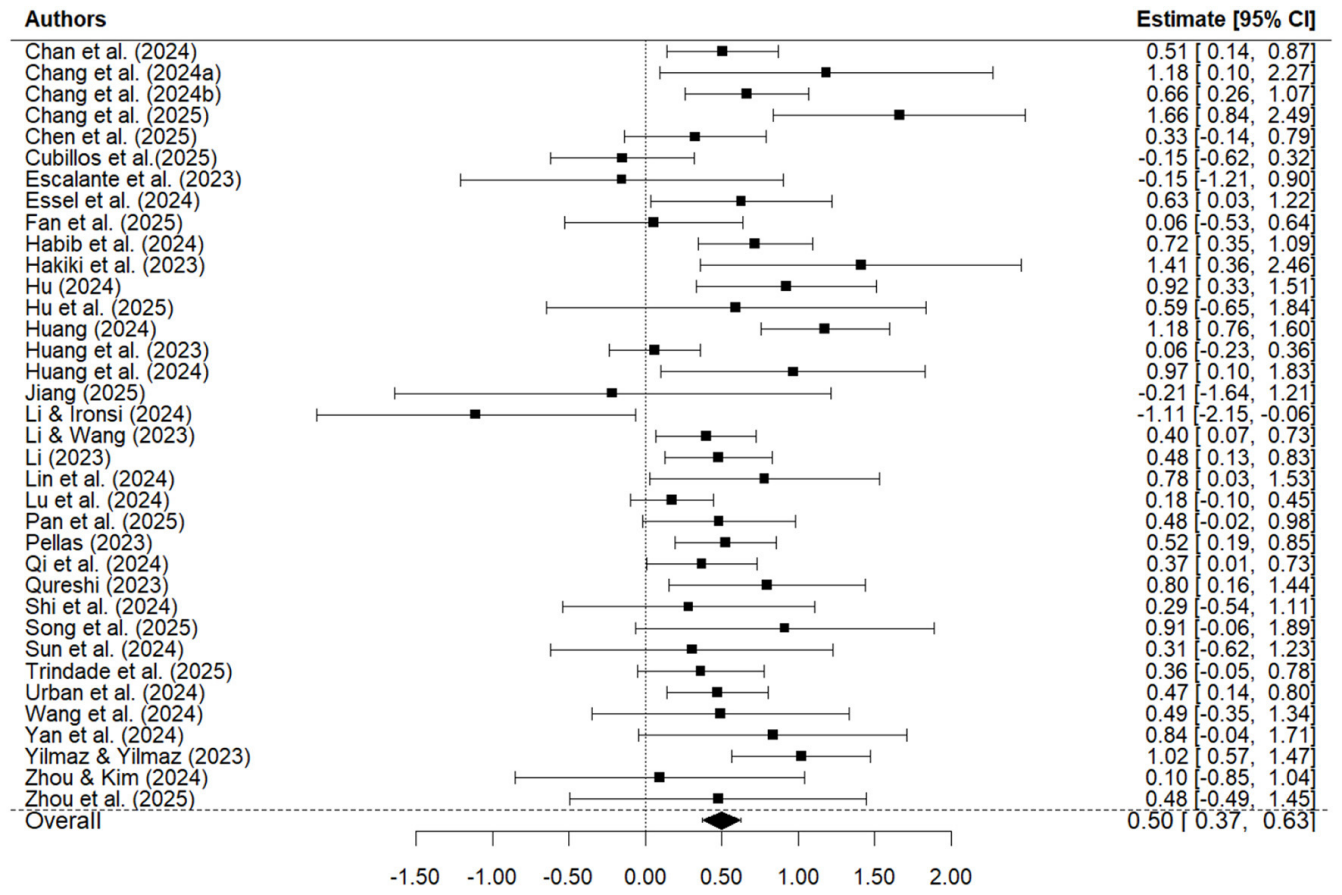
Study-level forest plot.

To provide an overview of study-level effects, Figure 3 presents a forest plot of the effect size estimate for each study together with its 95% confidence interval. The plot illustrates both the variability across studies and the consistency of the overall positive effect, as represented by the pooled diamond at the bottom.

### 4.2 Publication bias and sensitivity analysis

**Figure 4:**
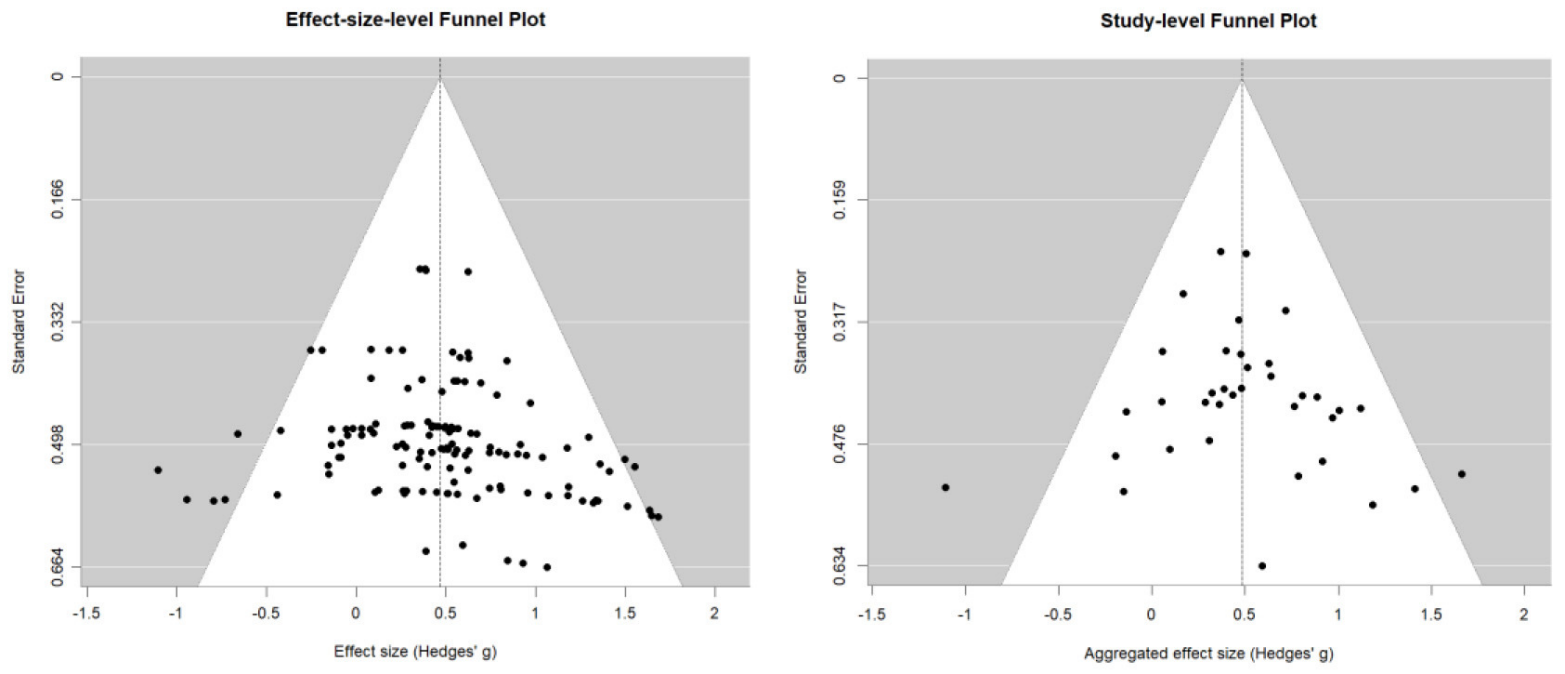
Funnel plot.

We assessed potential publication bias using a funnel plot and Egger's regression test, with the results shown in Figure 4. At the effect-size level, the funnel plot showed an approximately symmetric distribution of effect sizes around the overall mean effect. Egger's test was not statistically significant (*t* = 1.53, *p* = 0.129, bias = 0.599, *SE* = 0.392), providing no clear evidence of funnel plot asymmetry. However, because this analysis was based on multiple dependent effect sizes from the same studies, the result should be interpreted with caution given the independence assumption underlying conventional Egger's test. To address this issue, we additionally examined publication bias at the study level by aggregating multiple effect sizes within each study into a single study-level effect size. As shown in Figure 4, the study-level funnel plot was also approximately symmetric. The corresponding Egger's test was not statistically significant (*z* = 0.71, *p* = 0.475), likewise indicating no clear evidence of funnel plot asymmetry. The aggregated study-level meta-analytic estimate was *g* = 0.483 (*SE* = 0.065, 95% CI [0.356, 0.610], *p* < 0.0001). Taken together, these results suggest that there was no clear evidence of publication bias.

To further examine the robustness of the findings, three complementary sensitivity analyses were conducted, addressing the potential influence of extreme effect sizes, analytic assumptions, and study quality, respectively.

First, we conducted an outlier analysis to examine whether the overall findings were driven by extreme effect sizes. Outliers were flagged when an individual effect size fell outside the 95% confidence interval of the overall effect (Viechtbauer and Cheung, 2010). This procedure identified several extreme effect sizes (e.g., *g* = −1.11 and *g* = 1.68). After careful examination of the original reports, no major methodological flaws were identified, and thus these values were retained. A sensitivity analysis excluding the outliers yielded g = 0.480, nearly identical to the original estimate, indicating that the overall effect size is robust against their inclusion.

Second, to assess the sensitivity of the results to analytic assumptions, the assumed pretest–posttest correlation was varied from 0.4 to 0.6. The overall effect size remained essentially unchanged (see in Table 5), and the statistical significance of the results was not materially affected. These findings suggest that the results were robust to different plausible values of the pretest–posttest correlation.

**Table 5:** Sensitivity analysis varying *r*_*pre*−*post*_.

*r*	*g*	95% CI	SE	*t*	*p*
0.4	0.499	[0.362, 0.636]	0.070	7.118	<0.0001
0.5	0.499	[0.374, 0.624]	0.064	7.797	<0.0001
0.6	0.499	[0.387, 0.611]	0.057	8.717	<0.0001

Third, to examine whether the findings were influenced by methodologically weaker evidence, effect sizes from low-quality studies were excluded. A total of 7 effect sizes from 4 primary studies were identified as low quality. After excluding these effect sizes, the overall effect size was g = 0.477 (SE = 0.068, 95% CI [0.343, 0.610], *p* < 0.0001), showing only a small difference from the main analysis (*g* = 0.499). This suggests that the findings were robust to the exclusion of low-quality studies.

### 4.3 Moderator analyses

To explore potential sources of heterogeneity, seven moderators were examined: discipline category, measurement tool, intervention duration, teaching method, learning task type, GenAI platform, and GenAI model version. Consistent with recommendations by Schwarzer et al. (2015), all subgroup analyses were based on at least k = 10 effect sizes, ensuring adequate statistical power. The full results are presented in Table 6. Categories with *k* < 10 are retained for completeness but are not interpreted in the subsequent discussion.

**Table 6:** Moderator analyses of the effects of GenAI tools on learning outcomes.

Moderator variable	*k*	Intercept/g (95% CI)	SE	*F*_(df1, df2)_	*p*	Level 2 variance	Level 3 variance
Discipline categories				*F*_(2,104)_ = 1.249	0.291	0	0.358
Humanities science	14	0.313 [−0.125, 0.752]	0.221				
Natural science	52	0.674 [0.392, 0.956] ^***^	0.132				
Social science	41	0.423 [0.142, 0.703]^**^	0.135				
Measurement tools				*F*_(2,129)_ = 0.185	0.831	0	0.276
Expert rating	24	0.443 [0.206, 0.680] ^***^	0.120				
Self-rated scale	88	0.508 [0.349, 0.668] ^***^	0.081				
Standardized testing	20	0.551 [0.247, 0.854] ^***^	0.154				
Intervention duration				*F*_(4,119)_ = 0.411	0.801	0	0.315
<1 week	22	0.485 [0.140, 0.830]^**^	0.174				
1–5 weeks	49	0.573 [0.329, 0.817] ^***^	0.123				
6–10 weeks	36	0.419 [0.132, 0.706] ^**^	0.145				
11–15 weeks	15	0.357 [−0.049, 0.763]	0.205				
>15 weeks	2	0.828 [−0.086, 1.741]	0.461				
Teaching method				*F*_(4,127)_ = 2.613	0.038	0	0.186
Blended learning	29	0.633 [0.387, 0.879] ^***^	0.124				
Collaborative learning	11	1.026 [0.613, 1.439] ^***^	0.209				
Inquiry learning	25	0.371 [0.112, 0.631] ^**^	0.131				
Personalized learning	34	0.351 [0.138, 0.564] ^**^	0.108				
Traditional teaching	33	0.470 [0.269, 0.671] ^***^	0.102				
Learning task type				*F*_(1,130)_ = 0.904	0.344	0	0.267
Knowledge acquisition	42	0.416 [0.198, 0.634] ^***^	0.110				
Problem resolution	90	0.546 [0.385, 0.706]^***^	0.081				
GAI platform				*F*_(1,130)_ = 0.347	0.557	0	0.266
ChatGPT	95	0.476 [0.323, 0.629] ^***^	0.077				
Other	37	0.560 [0.320, 0.801] ^***^	0.121				
GAI model version				*F*_(1,65)_ = 1.206	0.276	0	0
ChatGPT-3.5	37	0.455 [0.307, 0.603] ^***^	0.074				
ChatGPT-4.0	30	0.329 [0.156, 0.503] ^***^	0.087				

The asterisks indicate levels of statistical significance: ^*^indicates *p* < 0.05, ^**^indicates *p* < 0.01, and ^***^indicates *p* < 0.001.

Among these moderators, teaching method was the only statistically significant moderator of GenAI's effect on learning outcomes (*F*_(4,127)_ = 2.613, *p* = 0.038). Subgroup analyses showed that collaborative learning yielded the largest effect size (*g* = 1.026), followed by blended learning (*g* = 0.633). Inquiry learning (*g* = 0.371), personalized learning (*g* = 0.351), and traditional teaching (*g* = 0.470) also showed positive but comparatively smaller effects. These findings suggest that GenAI tends to be most effective when embedded in interactive or integrative pedagogies, especially those involving peer collaboration.

The remaining six moderators were not statistically significant. Nevertheless, some patterns are noteworthy. For discipline category, natural sciences (*g* = 0.674) showed moderately larger effects than social sciences (*g* = 0.423) or humanities (*g* = 0.313). For measurement tools, standardized testing (*g* = 0.551) produced higher estimates than self-report scales (*g* = 0.508) or expert ratings (*g* = 0.443). Differences across intervention duration were minimal and inconsistent. Regarding technology-related moderators, both ChatGPT (*g* = 0.476) and other GenAI tools (*g* = 0.560) produced comparable effects, and the comparison between ChatGPT-3.5 (*g* = 0.455) and GPT-4.0 (*g* = 0.329) revealed no significant differences. Finally, for learning task type, problem resolution (*g* = 0.546) showed higher effects than knowledge acquisition (*g* = 0.416), suggesting a possible advantage when GenAI supports problem-solving activities. Taken together, these results indicate that while most study- or technology-level characteristics did not significantly alter the impact of GenAI, instructional method plays a central role in shaping its effectiveness in higher education.

## 5 Discussion and implications

Across 36 studies (132 effects), GenAI shows a moderate and significant positive association with learning outcomes in higher education (*g* = 0.499), consistent with Sun and Zhou's (2024) comparable estimate (*g* = 0.533). By distinguishing outcome categories, this study finds the strongest effects for understanding, creative, cognitive, and higher-order learning, moderate effects for dispositions, and smaller effects for attainments, with limited evidence for membership or inclusion or self-worth and for using or application outcomes due to sparse data. Rather than indicating a uniform benefit across all domains, these patterns suggest that GenAI is especially effective when learning outcomes depend on meaning-making, idea generation, explanation, critique, and revision rather than on the reproduction of fixed content alone. These patterns can be interpreted through three complementary mechanisms. The ICAP framework accounts for the largest gains in higher-order domains, since GenAI supports constructive and interactive engagement such as generating alternatives, critiquing responses, and refining ideas (Chi and Wylie, 2014). In other words, GenAI appears to improve learning primarily by moving students beyond passive reception toward deeper forms of cognitive engagement, and these processes are more directly connected to understanding, creative performance, and higher-order learning than to narrowly defined attainment measures. Dispositional improvements align with feedback-and-motivation explanations, as timely, targeted feedback enhances competence and autonomy in line with formative feedback research and self-determination theory (Hattie and Timperley, 2007; Shute, 2008; Ryan and Deci, 2000). This suggests that GenAI may strengthen learners' confidence and engagement not automatically, but when its feedback functions as a supportive scaffold that increases perceived competence and autonomy. Smaller attainment effects reflect assessment misalignment, where recall- and procedure-focused evaluations underestimate reasoning and integrative learning, consistent with concerns about constructive misalignment (Biggs, 1996; Pellegrino et al., 2001). Viewed together, these findings form a GenAI–Learning Alignment Perspective, indicating that GenAI's benefits depend on the alignment between engagement processes, learner needs, and assessment practices, or more specifically, on whether the cognitive demands of the task, the depth of learner engagement, and the forms of assessment are aligned in ways that allow GenAI-supported learning to be fully realized.

Teaching method was the only significant moderator, reaffirming that pedagogy rather than media determines learning outcomes (Clark, 1983, 1994). More importantly, this finding suggests that GenAI does not improve learning simply by being present; rather, its educational value is realized through the pedagogical structures that shape how learners interact with it. Collaborative learning showed the strongest effects, consistent with the ICAP framework, as interactive engagement through explanation, critique, and co-revision promotes iterative cycles of generation, evaluation, and refinement that deepen reasoning (Chi and Wylie, 2014). In collaborative settings, GenAI is more likely to become a shared object of discussion, challenge, and co-construction rather than merely an answer provider, which helps explain the larger observed gains. Blended learning also facilitated constructive engagement across preparation, in-class work, and review, though its distributed structure produced smaller gains. This may be because blended designs support sustained constructive engagement across phases of learning, but often provide fewer opportunities for intensive peer interaction than collaborative approaches. Inquiry, personalized, and teacher-centered approaches offered fewer opportunities for structured collaboration with GenAI, which aligns with concerns about reduced productive struggle when scaffolding is limited (Kirschner et al., 2006). In such contexts, GenAI may be more easily used for answer substitution or cognitive offloading than for shared reasoning and idea refinement, which may weaken its contribution to deeper learning. These findings extend meta-analytic evidence by clarifying GenAI's differentiated influence across learning dimensions within the LOTG framework (James and Brown, 2005) and by demonstrating that instructional design shapes its value. They also indicate that collaborative and blended designs, assessment practices that capture higher-order outcomes (Jisc, 2024a,b), and teacher professional development to enhance GenAI competence (Moorhouse et al., 2024) are central to maximizing benefits. Ethical and pedagogical guidance frameworks (UNESCO, 2023), learner support expectations (Jisc, 2025), and evolving competencies for life and work in the GenAI era (OECD, 2025) further underscore the need for alignment between GenAI use and institutional structures.

Across other moderators, including discipline category, measurement tool, intervention duration, learning task type, GenAI platform, and model version, no statistically significant moderating effects were detected, consistent with previous meta-analyses (Sun and Zhou, 2024; Zhu et al., 2025). Although small descriptive differences appeared across disciplines, assessment tools, or intervention duration were not significant, these differences were not statistically significant, suggesting that GenAI's effects did not vary markedly across the observed subgroups in the present dataset. Learning task type likewise showed limited evidence of moderation. Problem resolution tasks yielded slightly higher estimates than knowledge acquisition tasks, a pattern tentatively consistent with cognitive load theory (Sweller, 1988) and expertise-reversal effects (Kalyuga et al., 2003), although the difference was small and not statistically significant. Platform comparisons also showed no reliable variation, which may reflect the shared underlying architecture of common LLM-based tools, although this interpretation remains tentative (Norman, 2013). Model version also did not moderate outcomes, even though ChatGPT-4 surpasses ChatGPT-3.5 on reasoning benchmarks (OpenAI, 2023; Nori et al., 2023), which may be because instructional tasks rarely required capabilities beyond those of earlier models. This finding is also consistent with broader evidence showing no significant differences across educational technologies (Russell, 2001; Means et al., 2013; Tamim et al., 2011). Together, these results suggest that teaching method may be a more consequential source of variation in GenAI's effectiveness than the other moderators examined. By contrast, task-, platform-, and model-related variables showed limited evidence of differential effects, suggesting that GenAI's educational impact depends more on how it is pedagogically organized than on these contextual or technical distinctions.

At the same time, these positive effects should not be interpreted as unconditional benefits. Emerging evidence and policy guidance suggest that GenAI may also introduce important contextual and ethical risks in higher education (UNESCO, 2023; OECD, 2026). When learners rely on GenAI to complete cognitively demanding tasks, improved task performance does not necessarily translate into durable learning, and excessive cognitive offloading may weaken metacognitive engagement and long-term skill development. In addition, because GenAI systems can generate biased, inaccurate, or authoritative-sounding responses, uncritical use may amplify misinformation and uneven learning opportunities. These concerns are especially salient in assessment: if tasks can be completed largely by GenAI, assessment validity and academic integrity may be undermined unless assessment design is revised accordingly. Relatedly, GenAI can blur boundaries of authorship, attribution, and acceptable assistance, making transparent disclosure and clear institutional guidance increasingly necessary. Therefore, the educational value of GenAI depends not only on pedagogical alignment, but also on safeguards for academic integrity, assessment validity, transparency, and responsible human oversight.

## 6 Conclusions and limitations

This study conducted a three-level meta-analysis and found a medium positive effect of GenAI on learning outcomes in higher education. Effects were strongest for understanding, creative and cognitive outcomes, and higher-order learning, moderate for dispositions, smaller for attainments, and insufficient for membership, inclusion, self-worth, and using outcomes. Teaching method was the only significant moderator, highlighting the central role of pedagogy in shaping GenAI's impact. The study also offers higher-education-specific evidence and extends the LOTG framework to GenAI, while providing practical insights for instructional design, assessment, teacher development, and institutional policy. These contributions collectively form the GenAI–Learning Alignment Perspective, which emphasizes that GenAI's educational value depends on its alignment with core elements of teaching and learning.

Limitations include the restricted database scope. Although this review searched major international and Chinese academic databases, it was limited to peer-reviewed studies published in English or Chinese and did not systematically include gray literature, which may have introduced publication and language bias and led to the omission of relevant evidence from other sources. In addition, the number of studies was limited in several outcome categories and moderator subgroups. Future research should draw on more diverse and longitudinal evidence and examine specific outcome domains such as motivation or critical thinking.

## Data Availability

The original contributions presented in the study are publicly available. These data can be found in Zenodo at: https://doi.org/10.5281/zenodo.19939415.
