# Supplementary material for: Exploring the effect of GenAI on learning outcomes in higher education: a three-level meta-analysis
Source: Front Psychol. 2026 May 15;17:1758670. doi: 10.3389/fpsyg.2026.1758670 (PMC13219050; doi:10.3389/fpsyg.2026.1758670)
Supplement: Supplementary file 1 [file Supplementary_file_1.docx]

**Appendix A. Full coding results**

| Authors | Learning outcomes | Discipline category | Measurement  tool | Intervention duration | Teaching method | Learning task type | GAI brand | GAI model version |
| --- | --- | --- | --- | --- | --- | --- | --- | --- |
| Li & Wang (2023) | Understanding/cognitive/creative | Natural | Expert rating | 1-5 weeks | Inquiry learning | Problem resolution | ChatGPT | ChatGPT 3.5 |
| Li & Wang (2023) | Higher-order learning | Natural | Self-rated scale | 1-5 weeks | Inquiry learning | Problem resolution | ChatGPT | ChatGPT 3.5 |
| Li & Wang (2023) | Higher-order learning | Natural | Self-rated scale | 1-5 weeks | Inquiry learning | Problem resolution | ChatGPT | ChatGPT 3.5 |
| Li & Wang (2023) | Higher-order learning | Natural | Self-rated scale | 1-5 weeks | Inquiry learning | Problem resolution | ChatGPT | ChatGPT 3.5 |
| Li & Wang (2023) | Dispositions | Natural | Self-rated scale | 1-5 weeks | Inquiry learning | Problem resolution | ChatGPT | ChatGPT 3.5 |
| Li & Wang (2023) | Dispositions | Natural | Self-rated scale | 1-5 weeks | Inquiry learning | Problem resolution | ChatGPT | ChatGPT 3.5 |
| Li & Wang (2023) | Dispositions | Natural | Self-rated scale | 1-5 weeks | Inquiry learning | Problem resolution | ChatGPT | ChatGPT 3.5 |
| Li & Wang (2023) | Dispositions | Natural | Self-rated scale | 1-5 weeks | Inquiry learning | Problem resolution | ChatGPT | ChatGPT 3.5 |
| Qi et al. (2024) | Higher-order learning | Mixed | Self-rated scale | Other | Traditional teaching | Knowledge acquisition | Other | Other |
| Qi et al. (2024) | Higher-order learning | Mixed | Self-rated scale | Other | Traditional teaching | Knowledge acquisition | Other | Other |
| Yan et al. (2024) | Attainments | Natural | Standardized testing | >15 weeks | Traditional teaching | Problem resolution | Other | Other |
| Yan et al. (2024) | Using | Natural | Standardized testing | >15 weeks | Traditional teaching | Problem resolution | Other | Other |
| Chang et al. (2024a) | Higher-order learning | Natural | Self-rated scale | <1 weeks | Traditional teaching | Problem resolution | ChatGPT | ChatGPT 3.5 |
| Chang et al. (2024b) | Membership/inclusion/self-worth | Natural | Self-rated scale | 1-5 weeks | Traditional teaching | Problem resolution | ChatGPT | ChatGPT 4.0 |
| Chang et al. (2024b) | Understanding/cognitive/creative | Natural | Self-rated scale | 1-5 weeks | Traditional teaching | Problem resolution | ChatGPT | ChatGPT 4.0 |
| Chang et al. (2024b) | Understanding/cognitive/creative | Natural | Self-rated scale | 1-5 weeks | Traditional teaching | Problem resolution | ChatGPT | ChatGPT 4.0 |
| Chang et al. (2024b) | Understanding/cognitive/creative | Natural | Self-rated scale | 1-5 weeks | Traditional teaching | Problem resolution | ChatGPT | ChatGPT 4.0 |
| Chang et al. (2024b) | Understanding/cognitive/creative | Natural | Self-rated scale | 1-5 weeks | Traditional teaching | Problem resolution | ChatGPT | ChatGPT 4.0 |
| Chang et al. (2024b) | Understanding/cognitive/creative | Natural | Self-rated scale | 1-5 weeks | Traditional teaching | Problem resolution | ChatGPT | ChatGPT 4.0 |
| Chen et al. (2025) | Attainments | Humanities | Self-rated scale | 1-5 weeks | Inquiry learning | Knowledge acquisition | ChatGPT | ChatGPT 4.0 |
| Chen et al. (2025) | Dispositions | Humanities | Self-rated scale | 1-5 weeks | Inquiry learning | Knowledge acquisition | ChatGPT | ChatGPT 4.0 |
| Chen et al. (2025) | Dispositions | Humanities | Self-rated scale | 1-5 weeks | Inquiry learning | Knowledge acquisition | ChatGPT | ChatGPT 4.0 |
| Chen et al. (2025) | Dispositions | Humanities | Self-rated scale | 1-5 weeks | Inquiry learning | Knowledge acquisition | ChatGPT | ChatGPT 4.0 |
| Chen et al. (2025) | Dispositions | Humanities | Self-rated scale | 1-5 weeks | Inquiry learning | Knowledge acquisition | ChatGPT | ChatGPT 4.0 |
| Escalante et al. (2023) | Attainments | Humanities | Expert rating | 6-10 weeks | Traditional teaching | Knowledge acquisition | ChatGPT | ChatGPT 4.0 |
| Essel et al. (2024) | Higher-order learning | Mixed | Self-rated scale | 11-15 weeks | Traditional teaching | Problem resolution | ChatGPT | Other |
| Essel et al. (2024) | Higher-order learning | Mixed | Self-rated scale | 11-15 weeks | Traditional teaching | Problem resolution | ChatGPT | Other |
| Fan et al. (2025) | Dispositions | Mixed | Self-rated scale | <1 weeks | Traditional teaching | Problem resolution | ChatGPT | ChatGPT 4.0 |
| Fan et al. (2025) | Dispositions | Mixed | Self-rated scale | <1 weeks | Traditional teaching | Problem resolution | ChatGPT | ChatGPT 4.0 |
| Fan et al. (2025) | Dispositions | Mixed | Self-rated scale | <1 weeks | Traditional teaching | Problem resolution | ChatGPT | ChatGPT 4.0 |
| Hu et al. (2025) | Attainments | Mixed | Expert rating | <1 weeks | Collaborative learning | Problem resolution | Other | Other |
| Hu (2024) | Higher-order learning | Social | Self-rated scale | 1-5 weeks | Collaborative learning | Problem resolution | Other | Other |
| Authors | Learning outcomes | Discipline category | Measurement  tool | Intervention duration | Teaching method | Learning task type | GAI brand | GAI model version |
| Hu (2024) | Dispositions | Social | Self-rated scale | 1-5 weeks | Collaborative learning | Problem resolution | Other | Other |
| Hu (2024) | Understanding/cognitive/creative | Social | Self-rated scale | 1-5 weeks | Collaborative learning | Problem resolution | Other | Other |
| Huang et al. (2023) | Attainments | Natural | Standardized testing | 6-10 weeks | Personalized learning | Knowledge acquisition | ChatGPT | Other |
| Huang et al. (2023) | Higher-order learning | Natural | Self-rated scale | 6-10 weeks | Personalized learning | Knowledge acquisition | ChatGPT | Other |
| Huang et al. (2023) | Higher-order learning | Natural | Self-rated scale | 6-10 weeks | Personalized learning | Knowledge acquisition | ChatGPT | Other |
| Huang et al. (2023) | Higher-order learning | Natural | Self-rated scale | 6-10 weeks | Personalized learning | Knowledge acquisition | ChatGPT | Other |
| Huang et al. (2023) | Higher-order learning | Natural | Self-rated scale | 6-10 weeks | Personalized learning | Knowledge acquisition | ChatGPT | Other |
| Huang et al. (2023) | Higher-order learning | Natural | Self-rated scale | 6-10 weeks | Personalized learning | Knowledge acquisition | ChatGPT | Other |
| Huang et al. (2023) | Higher-order learning | Natural | Self-rated scale | 6-10 weeks | Personalized learning | Knowledge acquisition | ChatGPT | Other |
| Huang et al. (2023) | Higher-order learning | Natural | Self-rated scale | 6-10 weeks | Personalized learning | Knowledge acquisition | ChatGPT | Other |
| Huang et al. (2023) | Higher-order learning | Natural | Self-rated scale | 6-10 weeks | Personalized learning | Knowledge acquisition | ChatGPT | Other |
| Huang et al. (2023) | Higher-order learning | Natural | Self-rated scale | 6-10 weeks | Personalized learning | Knowledge acquisition | ChatGPT | Other |
| Huang (2024) | Understanding/cognitive/creative | Natural | Expert rating | 6-10 weeks | Collaborative learning | Problem resolution | Other | Other |
| Huang (2024) | Understanding/cognitive/creative | Natural | Expert rating | 6-10 weeks | Collaborative learning | Problem resolution | Other | Other |
| Huang (2024) | Understanding/cognitive/creative | Natural | Expert rating | 6-10 weeks | Collaborative learning | Problem resolution | Other | Other |
| Huang (2024) | Understanding/cognitive/creative | Natural | Expert rating | 6-10 weeks | Collaborative learning | Problem resolution | Other | Other |
| Huang (2024) | Dispositions | Natural | Self-rated scale | 6-10 weeks | Collaborative learning | Problem resolution | Other | Other |
| Huang (2024) | Dispositions | Natural | Self-rated scale | 6-10 weeks | Collaborative learning | Problem resolution | Other | Other |
| Huang (2024) | Higher-order learning | Natural | Self-rated scale | 6-10 weeks | Collaborative learning | Problem resolution | Other | Other |
| Huang et al. (2024) | Attainments | Social | Standardized testing | 1-5 weeks | Inquiry learning | Knowledge acquisition | Other | Other |
| Jiang (2025) | Attainments | Social | Expert rating | 6-10 weeks | Personalized learning | Knowledge acquisition | Other | Other |
| Jiang (2025) | Attainments | Social | Expert rating | 6-10 weeks | Personalized learning | Problem resolution | Other | Other |
| Li (2023) | Understanding/cognitive/creative | Social | Expert rating | 6-10 weeks | Blended learning | Problem resolution | ChatGPT | ChatGPT 3.5 |
| Li (2023) | Dispositions | Social | Self-rated scale | 6-10 weeks | Blended learning | Problem resolution | ChatGPT | ChatGPT 3.5 |
| Li (2023) | Dispositions | Social | Self-rated scale | 6-10 weeks | Blended learning | Problem resolution | ChatGPT | ChatGPT 3.5 |
| Li (2023) | Dispositions | Social | Self-rated scale | 6-10 weeks | Blended learning | Problem resolution | ChatGPT | ChatGPT 3.5 |
| Li (2023) | Dispositions | Social | Self-rated scale | 6-10 weeks | Blended learning | Problem resolution | ChatGPT | ChatGPT 3.5 |
| Li (2023) | Dispositions | Social | Self-rated scale | 6-10 weeks | Blended learning | Problem resolution | ChatGPT | ChatGPT 3.5 |
| Li (2023) | Higher-order learning | Social | Self-rated scale | 6-10 weeks | Blended learning | Problem resolution | ChatGPT | ChatGPT 3.5 |
| Lu et al. (2024) | Dispositions | Social | Self-rated scale | 1-5 weeks | Traditional teaching | Problem resolution | ChatGPT | ChatGPT 4.0 |
| Lu et al. (2024) | Higher-order learning | Social | Self-rated scale | 1-5 weeks | Traditional teaching | Problem resolution | ChatGPT | ChatGPT 4.0 |
| Lu et al. (2024) | Higher-order learning | Social | Self-rated scale | 1-5 weeks | Traditional teaching | Problem resolution | ChatGPT | ChatGPT 4.0 |
| Lu et al. (2024) | Higher-order learning | Social | Self-rated scale | 1-5 weeks | Traditional teaching | Problem resolution | ChatGPT | ChatGPT 4.0 |
| Lu et al. (2024) | Higher-order learning | Social | Self-rated scale | 1-5 weeks | Traditional teaching | Problem resolution | ChatGPT | ChatGPT 4.0 |
| Authors | Learning outcomes | Discipline category | Measurement  tool | Intervention duration | Teaching method | Learning task type | GAI brand | GAI model version |
| Lu et al. (2024) | Higher-order learning | Social | Self-rated scale | 1-5 weeks | Traditional teaching | Problem resolution | ChatGPT | ChatGPT 4.0 |
| Lu et al. (2024) | Higher-order learning | Social | Self-rated scale | 1-5 weeks | Traditional teaching | Problem resolution | ChatGPT | ChatGPT 4.0 |
| Pan et al. (2025) | Higher-order learning | Humanities | Self-rated scale | 11-15 weeks | Personalized learning | Knowledge acquisition | ChatGPT | ChatGPT 3.5 |
| Pan et al. (2025) | Higher-order learning | Humanities | Self-rated scale | 11-15 weeks | Personalized learning | Knowledge acquisition | ChatGPT | ChatGPT 3.5 |
| Pan et al. (2025) | Higher-order learning | Humanities | Self-rated scale | 11-15 weeks | Personalized learning | Knowledge acquisition | ChatGPT | ChatGPT 3.5 |
| Pan et al. (2025) | Higher-order learning | Humanities | Self-rated scale | 11-15 weeks | Personalized learning | Knowledge acquisition | ChatGPT | ChatGPT 3.5 |
| Trindade et al. (2025) | Understanding/cognitive/creative | Social | Self-rated scale | 11-15 weeks | Inquiry learning | Problem resolution | ChatGPT | ChatGPT 3.5 |
| Trindade et al. (2025) | Understanding/cognitive/creative | Social | Self-rated scale | 11-15 weeks | Inquiry learning | Problem resolution | ChatGPT | ChatGPT 3.5 |
| Trindade et al. (2025) | Understanding/cognitive/creative | Social | Self-rated scale | 11-15 weeks | Inquiry learning | Problem resolution | ChatGPT | ChatGPT 3.5 |
| Trindade et al. (2025) | Understanding/cognitive/creative | Social | Self-rated scale | 11-15 weeks | Inquiry learning | Problem resolution | ChatGPT | ChatGPT 3.5 |
| Trindade et al. (2025) | Understanding/cognitive/creative | Social | Self-rated scale | 11-15 weeks | Inquiry learning | Problem resolution | ChatGPT | ChatGPT 3.5 |
| Trindade et al. (2025) | Understanding/cognitive/creative | Social | Self-rated scale | 11-15 weeks | Inquiry learning | Problem resolution | ChatGPT | ChatGPT 3.5 |
| Trindade et al. (2025) | Understanding/cognitive/creative | Social | Self-rated scale | 11-15 weeks | Inquiry learning | Problem resolution | ChatGPT | ChatGPT 3.5 |
| Wang et al. (2024) | Attainments | Humanities | Expert rating | 6-10 weeks | Traditional teaching | Knowledge acquisition | Other | Other |
| Wang et al. (2024) | Attainments | Humanities | Expert rating | 6-10 weeks | Traditional teaching | Knowledge acquisition | Other | Other |
| Wang et al. (2024) | Membership/inclusion/self-worth | Humanities | Self-rated scale | 6-10 weeks | Traditional teaching | Knowledge acquisition | Other | Other |
| Zhou et al. (2025) | Attainments | Mixed | Standardized testing | 6-10 weeks | Blended learning | Knowledge acquisition | ChatGPT | ChatGPT 4.0 |
| Zhou et al. (2025) | Attainments | Mixed | Standardized testing | 6-10 weeks | Blended learning | Knowledge acquisition | ChatGPT | ChatGPT 4.0 |
| Zhou et al. (2025) | Attainments | Mixed | Standardized testing | 6-10 weeks | Blended learning | Knowledge acquisition | ChatGPT | ChatGPT 4.0 |
| Zhou & Kim (2024) | Attainments | Humanities | Expert rating | 6-10 weeks | Traditional teaching | Knowledge acquisition | ChatGPT | ChatGPT 4.0 |
| Chan et al. (2024) | Attainments | Mixed | Expert rating | <1 weeks | Personalized learning | Problem resolution | ChatGPT | ChatGPT 3.5 |
| Chan et al. (2024) | Dispositions | Mixed | Self-rated scale | <1 weeks | Personalized learning | Problem resolution | ChatGPT | ChatGPT 3.5 |
| Chang et al. (2025) | Dispositions | Natural | Self-rated scale | 1-5 weeks | Personalized learning | Knowledge acquisition | ChatGPT | Other |
| Chang et al. (2025) | Membership/inclusion/self-worth | Natural | Self-rated scale | 1-5 weeks | Personalized learning | Knowledge acquisition | ChatGPT | Other |
| Cubillos et al.(2025) | Attainments | Natural | Standardized testing | 1-5 weeks | Personalized learning | Problem resolution | Other | Other |
| Cubillos et al.(2025) | Dispositions | Natural | Standardized testing | 1-5 weeks | Personalized learning | Problem resolution | Other | Other |
| Cubillos et al.(2025) | Dispositions | Natural | Standardized testing | 1-5 weeks | Personalized learning | Problem resolution | Other | Other |
| Cubillos et al.(2025) | Dispositions | Natural | Standardized testing | 1-5 weeks | Personalized learning | Problem resolution | Other | Other |
| Cubillos et al.(2025) | Dispositions | Natural | Standardized testing | 1-5 weeks | Personalized learning | Problem resolution | Other | Other |
| Cubillos et al.(2025) | Dispositions | Natural | Standardized testing | 1-5 weeks | Personalized learning | Problem resolution | Other | Other |
| Habib et al. (2024) | Higher-order learning | Mixed | Expert rating | 1-5 weeks | Blended learning | Problem resolution | ChatGPT | Other |
| Habib et al. (2024) | Higher-order learning | Mixed | Expert rating | 1-5 weeks | Blended learning | Problem resolution | ChatGPT | Other |
| Habib et al. (2024) | Higher-order learning | Mixed | Expert rating | 1-5 weeks | Blended learning | Problem resolution | ChatGPT | Other |
| Authors | Learning outcomes | Discipline category | Measurement  tool | Intervention duration | Teaching method | Learning task type | GAI brand | GAI model version |
| Habib et al. (2024) | Higher-order learning | Mixed | Expert rating | 1-5 weeks | Blended learning | Problem resolution | ChatGPT | Other |
| Li & Ironsi (2024) | Attainments | Social | Expert rating | 11-15 weeks | Inquiry learning | Knowledge acquisition | ChatGPT | Other |
| Lin et al. (2024) | Using | Other | Standardized testing | 6-10 weeks | Inquiry learning | Problem resolution | ChatGPT | Other |
| Lin et al. (2024) | Higher-order learning | Other | Self-rated scale | 6-10 weeks | Inquiry learning | Problem resolution | ChatGPT | Other |
| Shi et al. (2024) | Attainments | Social | Standardized testing | Other | Inquiry learning | Knowledge acquisition | ChatGPT | ChatGPT 3.5 |
| Song et al. (2025) | Higher-order learning | Social | Expert rating | 11-15 weeks | Personalized learning | Problem resolution | Other | Other |
| Urban et al. (2024) | Understanding/cognitive/creative | Mixed | Self-rated scale | <1 weeks | Personalized learning | Problem resolution | ChatGPT | ChatGPT 3.5 |
| Urban et al. (2024) | Understanding/cognitive/creative | Mixed | Self-rated scale | <1 weeks | Personalized learning | Problem resolution | ChatGPT | ChatGPT 3.5 |
| Urban et al. (2024) | Higher-order learning | Mixed | Expert rating | <1 weeks | Personalized learning | Problem resolution | ChatGPT | ChatGPT 3.5 |
| Urban et al. (2024) | Higher-order learning | Mixed | Expert rating | <1 weeks | Personalized learning | Problem resolution | ChatGPT | ChatGPT 3.5 |
| Urban et al. (2024) | Higher-order learning | Mixed | Expert rating | <1 weeks | Personalized learning | Problem resolution | ChatGPT | ChatGPT 3.5 |
| Urban et al. (2024) | Dispositions | Mixed | Self-rated scale | <1 weeks | Personalized learning | Problem resolution | ChatGPT | ChatGPT 3.5 |
| Yilmaz & Yilmaz (2023) | Higher-order learning | Natural | Self-rated scale | 1-5 weeks | Blended learning | Knowledge acquisition | ChatGPT | Other |
| Yilmaz & Yilmaz (2023) | Higher-order learning | Natural | Self-rated scale | 1-5 weeks | Blended learning | Knowledge acquisition | ChatGPT | Other |
| Yilmaz & Yilmaz (2023) | Higher-order learning | Natural | Self-rated scale | 1-5 weeks | Blended learning | Knowledge acquisition | ChatGPT | Other |
| Yilmaz & Yilmaz (2023) | Higher-order learning | Natural | Self-rated scale | 1-5 weeks | Blended learning | Knowledge acquisition | ChatGPT | Other |
| Yilmaz & Yilmaz (2023) | Dispositions | Natural | Self-rated scale | 1-5 weeks | Blended learning | Knowledge acquisition | ChatGPT | Other |
| Yilmaz & Yilmaz (2023) | Dispositions | Natural | Self-rated scale | 1-5 weeks | Blended learning | Knowledge acquisition | ChatGPT | Other |
| Hakiki et al. (2023) | Attainments | Social | Standardized testing | Other | Traditional teaching | Knowledge acquisition | ChatGPT | Other |
| Pellas 2023 | Understanding/cognitive/creative | Social | Self-rated scale | <1 weeks | Blended learning | Problem resolution | Other | Other |
| Pellas (2023) | Understanding/cognitive/creative | Social | Self-rated scale | <1 weeks | Blended learning | Problem resolution | Other | Other |
| Pellas (2023) | Understanding/cognitive/creative | Social | Self-rated scale | <1 weeks | Blended learning | Problem resolution | Other | Other |
| Pellas (2023) | Understanding/cognitive/creative | Social | Self-rated scale | <1 weeks | Blended learning | Problem resolution | Other | Other |
| Pellas (2023) | Understanding/cognitive/creative | Social | Self-rated scale | <1 weeks | Blended learning | Problem resolution | Other | Other |
| Pellas (2023) | Dispositions | Social | Self-rated scale | <1 weeks | Blended learning | Problem resolution | Other | Other |
| Pellas (2023) | Dispositions | Social | Self-rated scale | <1 weeks | Blended learning | Problem resolution | Other | Other |
| Pellas (2023) | Dispositions | Social | Self-rated scale | <1 weeks | Blended learning | Problem resolution | Other | Other |
| Pellas (2023) | Dispositions | Social | Self-rated scale | <1 weeks | Blended learning | Problem resolution | Other | Other |
| Qureshi (2023) | Attainments | Natural | Standardized testing | Other | Traditional teaching | Problem resolution | ChatGPT | ChatGPT 4.0 |
| Qureshi (2023) | Attainments | Natural | Standardized testing | Other | Traditional teaching | Problem resolution | ChatGPT | ChatGPT 4.0 |
| Qureshi (2023) | Attainments | Natural | Standardized testing | Other | Traditional teaching | Problem resolution | ChatGPT | ChatGPT 4.0 |
| Qureshi (2023) | Attainments | Natural | Standardized testing | Other | Traditional teaching | Problem resolution | ChatGPT | ChatGPT 4.0 |
| Sun et al. (2024) | Using | Social | Expert rating | 1-5 weeks | Personalized learning | Problem resolution | ChatGPT | ChatGPT 3.5 |
